# Spatiotemporal Rule of Heat Transfer on a Soil/Finned Tube Interface

**DOI:** 10.3390/s19051159

**Published:** 2019-03-07

**Authors:** Yongsheng Huang, Wenbin Li, Daochun Xu, Yafeng Wu

**Affiliations:** Key Lab of State Forestry Administration on Forestry Equipment and Automation, School of Technology, Beijing Forestry University, Beijing 100083, China; huangyongsheng@bjfu.edu.cn (Y.H.); xdl_wyf314@163.com (Y.W.)

**Keywords:** environmental micro-energy, heat transfer, spatiotemporal, finned tube, soil

## Abstract

To efficiently harvest environmental micro-energy from shallow soil, simulated analysis, theoretical arithmetic and experimental verification are performed to explore the spatiotemporal rules of heat transfer on a soil/finned tube interface. Simulations are carried out for 36 types of different working conditions, and the empirical formulas for temperature and heat flux are obtained. The temperature and heat flux can be calculated using the formulas if the soil temperature, soil moisture content and finned tube initial temperature are known. The simulations also show that the highest heat flux can reach approximately 0.30 mW/mm^2^, and approximately 1507.96 mW of energy can be harvested through the finned tube. Theoretical arithmetic indicates that the heat transfer rate of the copper finned tube is 76.77% higher than that of the bare tube, the highest rate obtained in any study to date. Results also show that the finned tube should be placed where the soil moisture is greater than 30% to get more heat from the soil. A field experiment is carried out in the city of Harbin in Northeast China, where a thermoelectric power generation device has been installed and temperature data have been monitored for a certain time. The results are in good agreement with those obtained from the simulation analysis. The heat transfer processes and heat transfer steady state on the soil/finned tube interface are revealed in this work and are of great importance for the use of geothermal energy.

## 1. Introduction

**Figure 1:**
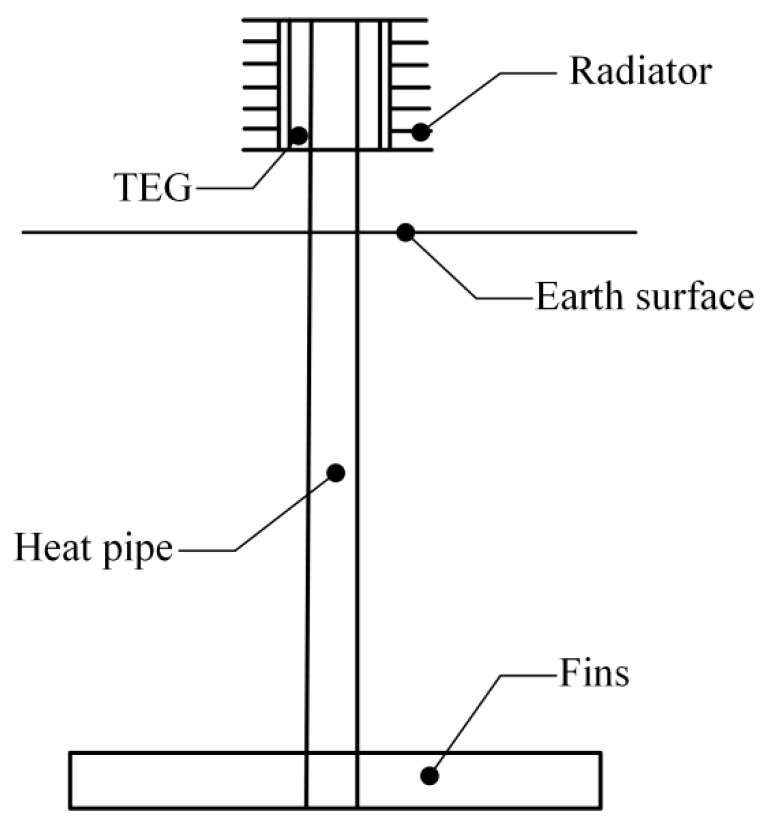
Core elements of thermoelectric power generation device.

Due to the special environment in remote forest areas, the question of how to power wireless sensors in a forest has been a challenge for scientists. Stevens [1] and Lawrence and Snyder [2] proposed a new method that uses the temperature difference between the forest soil and air to generate electricity based on the Seebeck effect. Zhang and Li [3,4] and Nuwayhid [5] also developed generation devices based on the Seebeck effect. As shown in Figure 1, one feasible scheme transmits heat from the shallow forest soil to the surface by a gravity-assisted heat pipe and generates electricity via thermoelectric power generation (TEG) [6,7]. To acquire as much heat and generate as much electricity as possible, a heat exchanger must be attached to the gravity-assisted heat pipe [3]. The spatiotemporal rule of heat transfer on the soil/finned tube interface during heat absorption is critical in the harvesting of environmental micro-energy from shallow soil, thus, this work focuses on this objective.

In previous work, researchers focused on the effects of certain parameters on the heat transfer from the heat exchanger or the performance of different fin shapes, with most of previous work focused on the ground source heat pump system (GSHP) and ground heat exchanger (GHE). Khalajzadeh et al. [8] also optimized the parameters of vertical ground heat exchangers and found that the inlet fluid temperature and pipe diameter significantly affected the heat transfer, while the burial depth was not as important. In their work, a group of optimal parameters was obtained by theoretical calculation as well. Other researchers have studied the effects of different numbers of fins and fin length in a radial finned tube buried in the soil [9]. Both computational fluid dynamics (CFD) simulations and field experiments were carried out, and the results showed that if the material was aluminium, the best two combinations were an 8-fin tube with a fin length of 30 mm and a 2-fin tube with a fin length of 80 mm.

Other researchers studied the temperature distribution in the soil surrounding the finned tubes. Kayaci and Demir [10] simulated the temperature distribution in the soil surrounding a GSHP system for 10 years and performed field experiments. Beier [11] developed a transient heat transfer model in a vertical borehole that could forecast the temperature profiles in the ground. He also deduced an energy equation that can solve the heat conduction equations for the ground in the vertical direction. Rouag et al. [12] studied the temperature in the soil to determine the optimum distance between heat exchangers using a newly developed semi-analytical method. Li et al. [13] established a multi-function ground-source heat pump system and performed simulations under the conditions of the middle and down-stream regions of the Yangtze River in China. In the simulation, the profile of the soil temperature surrounding the U-tube heat exchangers was clearly shown under different operating conditions. To improve the performance of the GSHP, Qi et al. [14] replaced common soil with three types of phase-change materials to backfill the borehole of the GHE and studied the effects of each. These results showed that PCM grouting materials had smaller thermal effect radii and behaved better than soil. Borinaga-Treviño et al. [15] also found that a cement-bentonite-graphite mix produced better transfer results, and in their work, the borehole thermal resistance and undisturbed vertical ground temperature profile were studied. The simulations in those research studies were carried out with the help of FLUENT [9,12,14,16,17] or MATLAB [10] software.

The core issue in this power generation scheme is ensuring that the temperature difference between the hot and cold sides of the TEG is as large as possible [5,18,19,20,21]. The cold side should be kept cool by the radiator, but the hot side of the TEG should be heated up as well. The gravity-assisted heat pipe has high thermal conductivity compared to other conventional metals, but its contact area with the soil is limited by the diameter, which is typically less than 40 mm. The references above demonstrate that the finned tube can extend the region of contact with the soil or other grouting material and greatly improve the thermal conduction. However, the questions of how the temperature profile changes when heat transfers out of soil, how the temperature is distributed once the heat transmission reaches a balance and how much heat can be obtained in a given environment are ambiguous. In this paper, simulations and theoretical arithmetic and field experiment are carried out to clarify the spatiotemporal rules of heat transfer on the soil/finned tube interface and the conducive environment for device placing, to determine what affects the energy harvesting and by how much can the finned tube increase the efficiency, and two empirical formulas are given for temperature and heat flux calculation.

## 2. Materials and Methods

### 2.1. Materials

**Figure 2:**
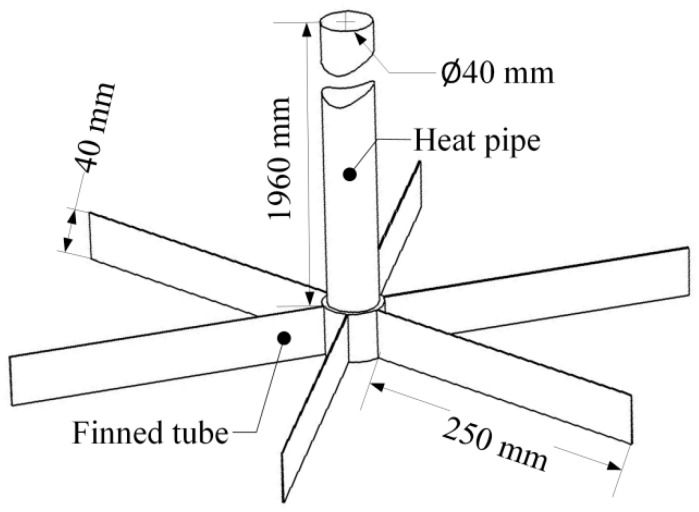
Finned tube attached to a heat pipe.

The length of the gravity-assisted heat pipe used in this project ranges from 2000 mm to 3500 mm [22] by 500 mm, but only one-fiftieth of the length is assigned to the evaporation end, another one-fiftieth is used in the condensation end, and the remainder of the pipe shell is adiabatic. In terms of manufacturing and cost, arranging thin fins on the copper tube and attaching them to the evaporation end of a gravity-assisted heat pipe is a rational approach. So, a copper tube with a length of 40 mm, inside diameter of 40 mm and outside diameter of 48 mm with thin fins (which have a length of 250 mm, width of 40 mm and thickness of 1 mm) was employed (Figure 2).

To facilitate the study, certain assumptions were applied in this finned tube model [16,23,24]:

- The soil surrounding the gravity-assisted heat pipe is isotropous, and the temperature distribution is homogeneous at a given depth;
- The thermal contact resistance at the interface is not considered;
- No internal heat is generated.

### 2.2. Set-up of the Model

**Figure 3:**
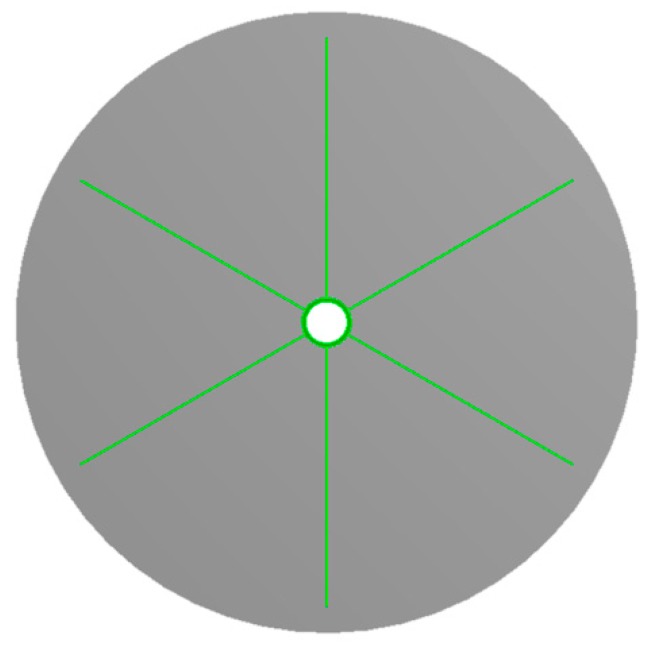
Two-dimensional finned tube model.

For a cylindrical heat exchanger with a uniform cross-sectional shape, the heat transfer law of each cross-section is equally representative, and the calculation of the two-dimensional model is simpler and faster, and the result is more accurate, so based on the simulations described above, a two-dimensional finned tube model (Figure 3) has been built [10,25,26], where the soil appears in grey, the finned tube in green, and the model’s thickness is 0 mm. The simulation is performed using the computational software ANSYS-Workbench 16.1 (ANSYS, Canonsburg, PA, USA). In order to get the changing processes and the end results, the simulations employed the steady-state thermal and transient thermal parameters in the toolbox and they were associated, whereby the steady-state thermal solution was used to provide the initial conditions for the transient thermal model. For the sake of establishing the appropriate soil radius, three simulations were carried out with the radii set at 300, 400 and 500 mm, but with the same type of mesh. The results showed no difference, indicating that the simulation domain radius can be set as 300 mm. There were 89,028 quadrilateral mesh elements in the simulation model and another simulation (using a radius of 300 mm and 120,166 elements) has been launched to verify that the result was independent of the mesh size, whereby the results showed the relative change rate was about 0.8%, less than our grid convergence index criterion of 1% which means the mesh size in our model was reasonable. Step time independence has been verified too, and the step time was set as 0.25, 0.5, 1.0 and 1.5 s and the results showed that temperature change was minute when the step time is shorter than 1.0 s, so the step time in our study was adopted as 1.0 s and 9000 steps were used in our simulations. Under the conditions described above, the residual of the model was less than 1 × 10^−4^.

According to the reports by Agrawal et al. [27] and Yang et al. [23], the thermal conductivity of the soil is related to its moisture content, so a new material library of soil was built in which the soil moisture (M) ranges from 10% to 35% in increments of 5%. All of the thermal conductivity, specific heat and density data acquired from our previous work are listed in Table 1, and the material of the finned tube is standard copper. The initial temperatures of the soil (*T_s_*) and finned tube (*T_f_*) were selected as 20, 15, and 10 °C (typically the temperature in shallow soil is between 0 and 20 °C) and 15, 10, and 5 °C (the air temperature is lower than that of soil in winter), respectively, with ranges (*T_s_–T_f_*) of 20–15 °C, 20–10 °C, 20–5 °C, 15–10 °C, 15–5 °C, and 10–5 °C, resulting in 36 types of operating conditions if moisture content was considered.

**Figure 4:**
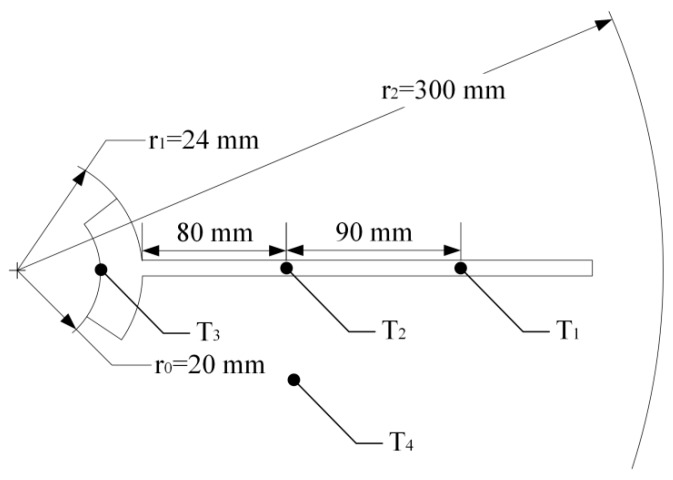
Map of temperature probe distribution.

Heat-conducting silicone grease was painted onto the contact area of the heat pipe and finned tube to improve its heat-conducting properties. The thermal conductivity of the heat-conducting silicone grease used in the experiments was 5.15 Wm^−1^ k^−1^, and considering the uneven application, the thermal conductivity in this component was set at 5 Wm^−1^ k^−1^. As shown in Figure 4, three temperature probes (*T*_1_, *T*_2_, and *T*_3_) were placed on the finned tube to detect the temperature changes in the fin, and another temperature probe (*T*_4_) were placed in the soil in addition to the temperature probe *T*_2_.

### 2.3. Mathematics Model

No currently available formula model is capable of calculating the temperature distribution in the soil surrounding the finned tube, as is considered in this work; therefore, the finned tube model is simplified as a cylinder for the numerical calculations. The differential equation of heat conduction is given in cylindrical coordinates as follows [28]:

(1) $$\rho c\frac{\partial T}{\partial \tau }=\frac{1}{r}\frac{\partial }{\partial r}\left(\lambda r\frac{\partial T}{\partial r}\right)+\frac{1}{r^{2}}\frac{\partial }{\partial \theta }\left(\lambda \frac{\partial T}{\partial \theta }\right)+\frac{\partial }{\partial z}\left(\lambda \frac{\partial T}{\partial z}\right)+q_{v}$$

where ρ is the density of the soil, c is the specific heat of the soil, T is the temperature of the soil, τ is the time, λ is the thermal conductivity of the soil, and $q_{v}$ is the heat produced by the soil per unit volume in unit time, and in this work, the value of $q_{v}$ is 0. Based on the assumptions in this work, when the steady state is reached, the equation can be written as follows:

(2) $$\frac{d}{dr}\left(r\frac{dT}{dr}\right)=0$$

Subsequently, the temperature distribution in the cylinder soil can be obtained as [28]:

(3) $$T=T_{a}-(T_{a}-T_{b})\frac{\ln\frac{r}{r_{1}}}{\ln\frac{r_{2}}{r_{1}}}$$

where *r*_1_ is the outside radius of the copper tube, *T_a_* is the temperature of the outward surface of copper tube (because no thermal contact resistance at the interface is considered, *T_a_* can also be viewed as the temperature of soil on this surface), *r*_2_ is the radius of the soil model, and *T_b_* is the temperature of the soil on this surface. If *r*_2_ is sufficiently large and the temperature on this surface changes minimally, then *T_b_* is assumed to be constant.

Our environmental micro-energy harvesting device or any other ground-source heat pump requires heat flux from the soil. According to Fourier’s law, the heat conduction quantity of the cylinder wall with length *L* is written as follows [28]:

(4) $$\Phi =-\lambda \frac{dT}{dr}(2\pi rL)$$

In this paper it can be written as:

(5) $$\Phi =\frac{T_{a}-T_{b}}{\frac{1}{2\pi \lambda L}\ln\frac{r_{2}}{r_{1}}}$$

## 3. Results and Discussion

**Figure 5:**
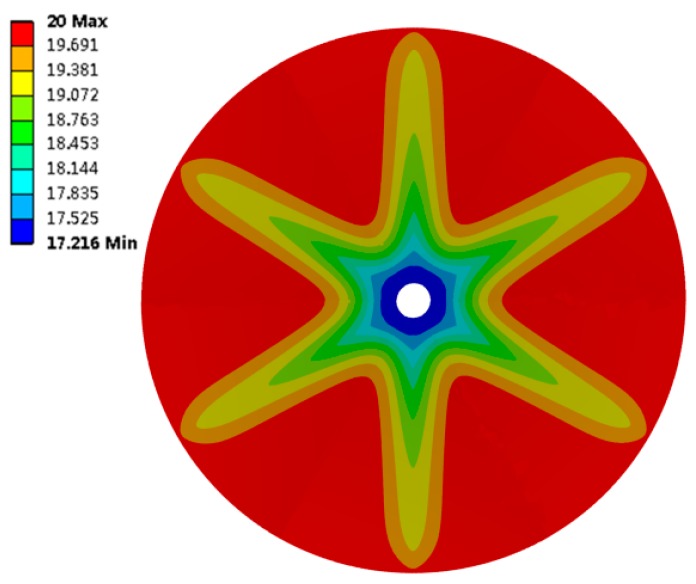
Temperature distribution in soil when *T_s_* is 20 °C, *T_f_* is 5 °C and soil moisture is 30%.

**Figure 6:**
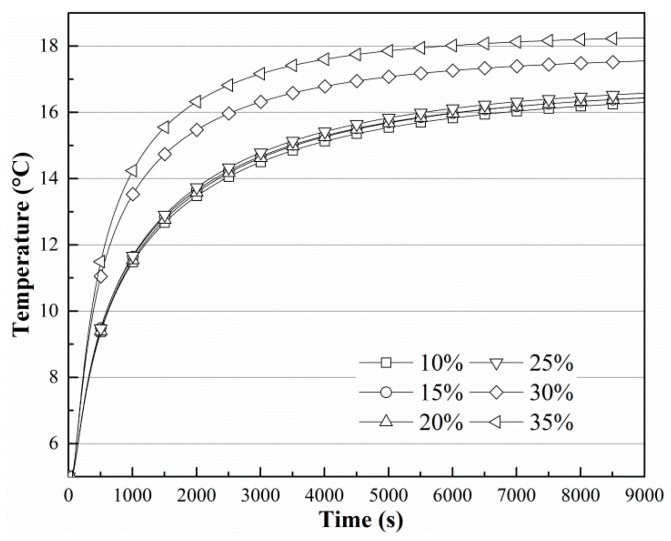
Temperature change in *T*_3_ when *T_s_* is 20 °C and *T_f_* is 5 °C.

**Figure 7:**
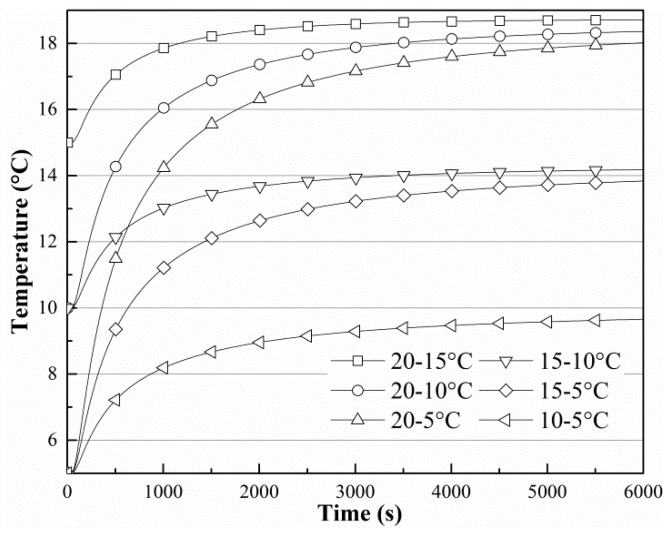
Temperature change in *T*_3_ when the soil moisture is 35%.

The transient thermal results have been studied. Figure 5 is the contour plot of the temperature in the soil, where we can see that the shape of the contour is similar to that of the finned tubes, and the temperature around the fins is lower. Figure 6 shows the change in *T*_3_ when the soil temperature is 20 °C and the finned tube temperature is 5 °C. Figure 7 shows the change in *T*_3_ when the soil moisture content is 35%. In Figure 6, the curves change with the same tendency, and curves with soil moisture contents below 30% change synchronously and they reach an equilibrium state after more than 9000 s. Curves with soil moisture content above 30% can reach the equilibrium state in about 6000 s, indicating that ground-source heat devices should be placed in locations where the moisture content is higher [27], and 30% is at least a threshold value. As shown in Figure 7, the environmental temperature affects the steady state only minimally. The temperatures at *T*_3_ all converge on the initial temperature of soil, and the heat conduction effect of the finned tube is good.

**Figure 8:**
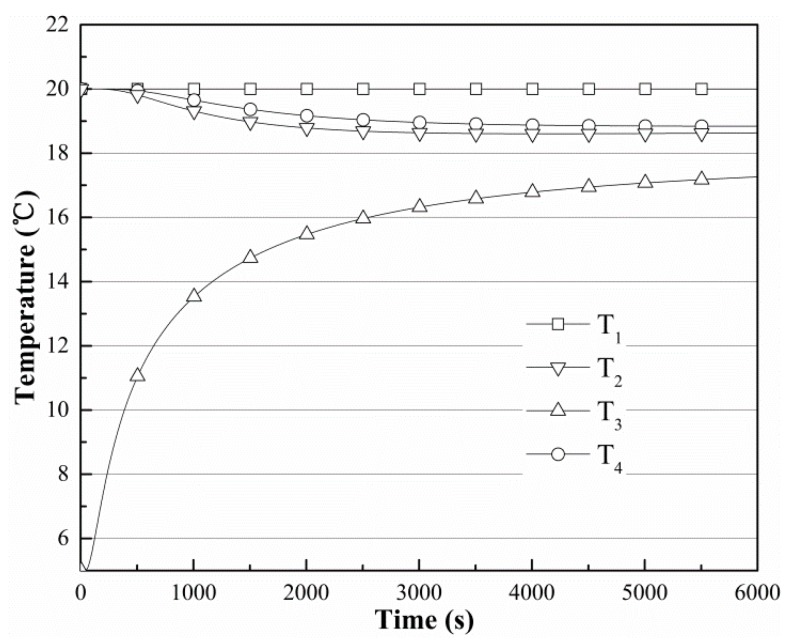
Temperature change when the soil moisture is 30%, *T_s_* is 20 °C, and *T_f_* is 5 °C.

The temperature change situations for four sites (*T*_1_, *T*_2_, *T*_3_, and *T*_4_) are presented in Figure 8, where the initial soil temperature is 20 °C, the moisture content is 30%, and the initial temperature of the finned tube is 5 °C. The temperatures at *T*_2_ and *T*_4_ change similarly, indicating that the temperature of the finned tube is more heavily influenced by the distance of the radius of the copper tube in this model. The temperature of *T*_1_ changes only slightly during the whole process, suggesting that the finned tube’s sphere of influence is limited in the surrounding soil.

**Figure 9:**
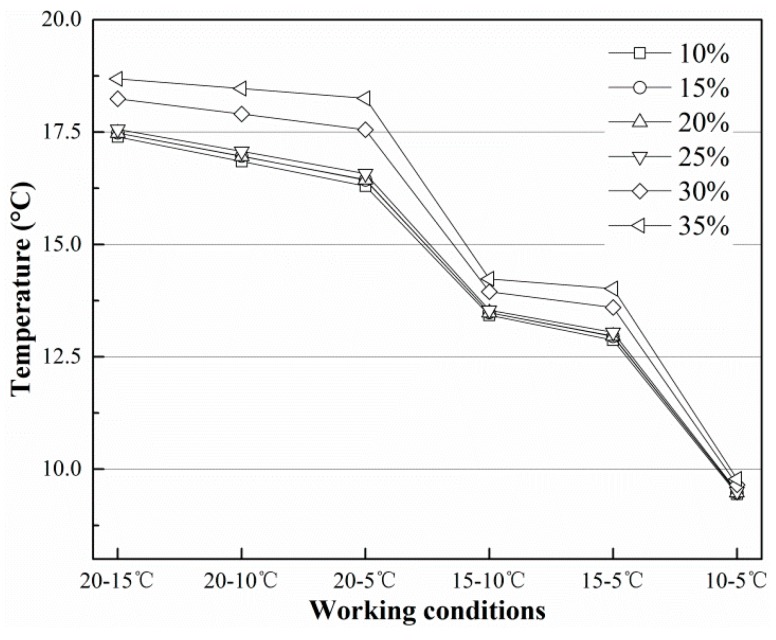
Distribution of *T*_3_ in different conditions in equilibrium state.

**Figure 10:**
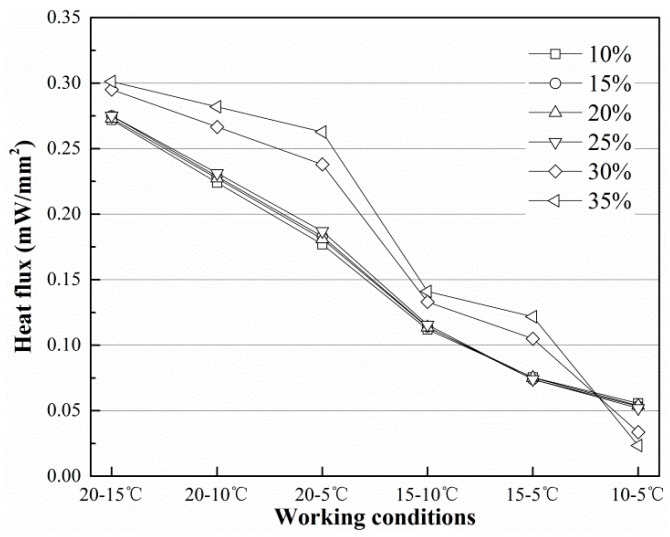
Heat flux through the finned tube in different conditions in equilibrium state.

Figure 9 and Figure 10 show the temperature at *T*_3_ and the heat flux on the finned tube in the final equilibrium state under different working conditions. In Figure 9 and Figure 10, the temperature and heat flux decrease as the initial temperature of the finned tube decreases, but the decrease is more rapid with decreases in the soil temperature. In general, the distance between curves tends to decrease, but the gap between the two curves increases when the initial temperature of the finned tube decreases while the initial temperature of the soil remains at the same level. According to Figure 10, the highest heat flux on the finned tube can reach approximately 0.30 mW/mm^2^, and at this rate, approximately 1507.96 mW of energy can be absorbed from the soil under the corresponding working conditions.

In the numerical calculations, because our model has been simplified as a hollow cylinder [12], the number obtained from Equation (5) only applies to the bare tube. The soil with a moisture content of 35% conducts approximately 2509.01 mW of heat, according to Equation (5). If we consider the bare tube, the largest heat exchange efficiency is approximately 0.34 [8], and approximately 853.06 mW of energy can be obtained through the bare tube.

A simulation for the bare tube was performed (the domain radius was 300 mm, the number of elements was 82,796 and step time was 1 s; a domain independence, grid independence and step independence tests were also performed), and the results compared with the calculated value. The simulation result shows that the highest heat flux is approximately 0.17 mW/mm^2^. A bare tube can harvest energy at a rate of 838.56 mW, the error between the simulated and calculated results is 1.70%, and the calculated result is approximate. From this perspective, the heat transfer rate for our finned tube (1507.96 mW) is 76.77% higher than that of the bare tube, which is 50% greater than in the work of Demir [9] and 7% more than in the work of Bouhacina [17]. This difference might be explained by three reasons:

(1) The material of the finned tube is aluminium in Demir’s work and polyethylene in Bouhacina’s, whereas it is copper in this work. The thermo-physical properties of copper are better than those of either aluminium or polyethylene;
(2) The fin length is 150 mm in Demir’s work, whereas it is 250 mm in this work. The area of the contact region is the smallest in Bouhacina’s internal finned tube;
(3) The moisture levels for soil are 15% in Demir’s work and 35% in this work, and a higher soil moisture content is associated with better thermal conductivity.

The data in Table 2 show the temperature data of *T*_3_ and the heat flux through the finned tube under different working conditions. According to the data and figures above, the final value of *T*_3_ and the heat flux are affected by *T_s_*, *T_f_* and soil moisture content (*M*). The empirical formulas are obtained through multiple linear regression analysis and multivariate nonlinear regression analysis, the temperature at *T*_3_ is written as follows:

(6) $$T_{3}=0.7370T_{s}+0.0882T_{f}+4.6255M+0.7055$$

The heat flux (*q*) is given as follows:

(7) $$\begin{array}{l} q=0.0016T_{s}{}^{2}+7.7025T_{f}{}^{2}+0.9479M^{2}\\ -0.0309T_{s}+0.0061T_{f}-0.2828M+0.1740 \end{array}$$

**Figure 11:**
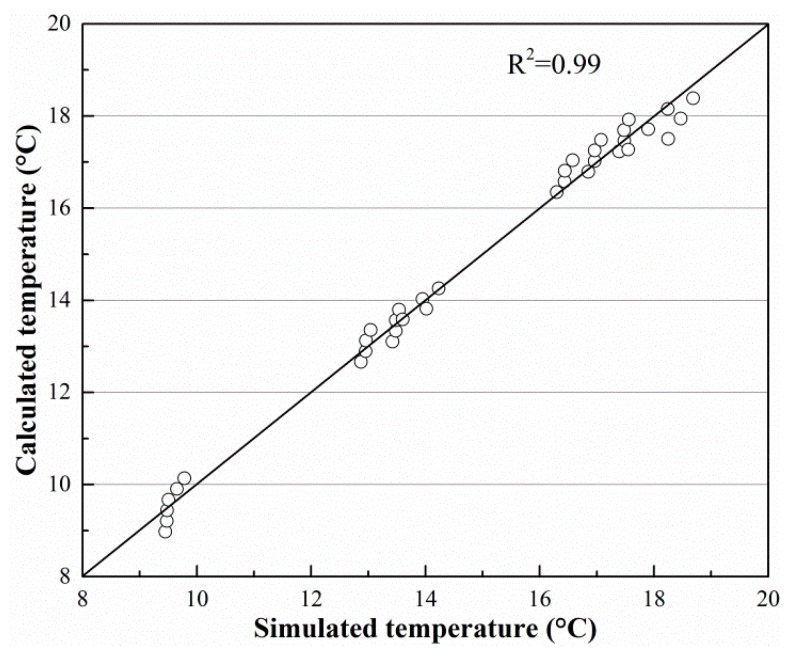
Imitative effect of Equation (6).

**Figure 12:**
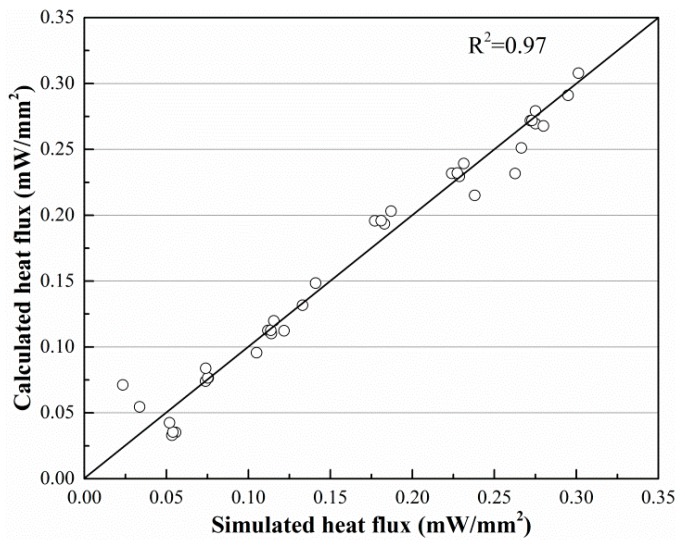
Imitative effect of Equation (7).

Figure 11 and Figure 12 show the imitative effect and determination coefficients of Equations (6) and (7). Both goodness of fit (R^2^) values are greater than 97%; thus, the equation analogies are satisfactory.

**Figure 13:**
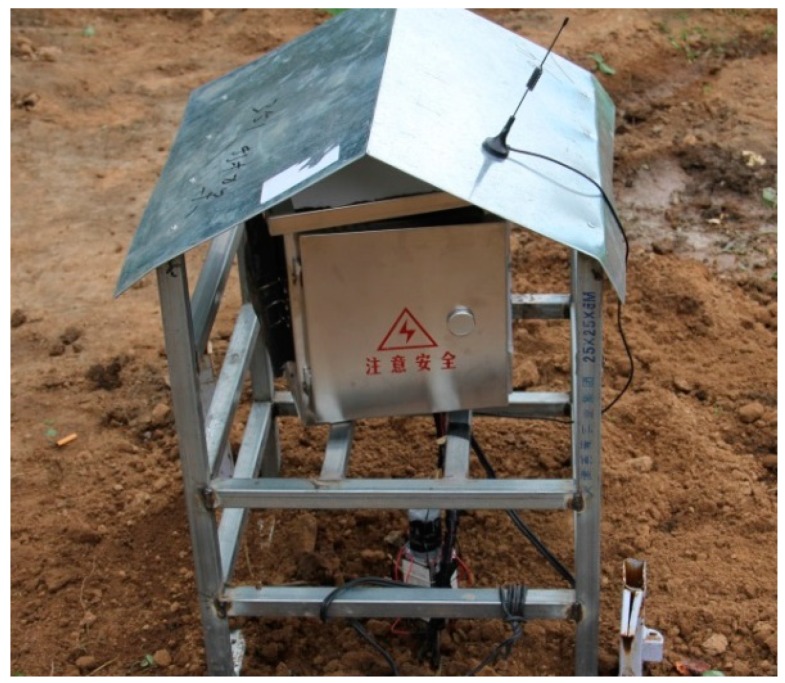
View of the field experiment device in Harbin.

**Figure 14:**
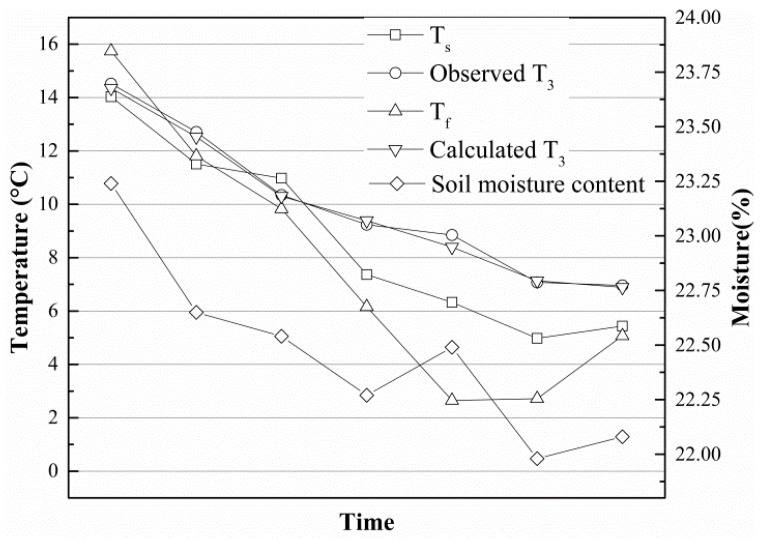
Observed and calculated temperature distribution on the finned tube in Harbin.

As shown in Figure 13, a field experiment was carried out in the city of Harbin (45°14′ N/126°12′ E), Heilongjiang Province, China. Just as shown in Figure 1, the core elements of this thermoelectric power generation device consist of a heat exchanger, gravity-assisted heat pipe, TEG and radiator. The gravity-assisted heat pipe used in this work was 40 mm in diameter and 3.5 m in length, the phase changeable working fluid is inorganic salt. Eight TEGs (TG12-6-02, Marlow Industries, Dallas, TX, USA) 43 mm in length, 40 mm in width and 3 mm in thickness were attached to the condensation end of the gravity-assisted heat pipe though a square outside and cylindrical inside copper block, and the cold side was cooled by eight finned plated radiators. Most of the thermoelectric power generation device was buried in a vertical hole, and only the condensation end of gravity-assisted heat pipe, TEGs and radiator were above ground. All contact surfaces were painted with thermally conductive silicone to improve the thermal conduction. The thermocouples and soil temperature and moisture content sensors (TM) were placed on the device and surrounding soil (Figure 4). These data and the generating capacity signal were transmitted to a networking platform by general packet radio service (GPRS). According to our study, the temperature on hot side of the TEG affected by soil temperature, air temperature and soil moisture, if the temperature difference between soil and air reach 20 °C, the temperature difference between hot and cold side of TEG is about 5 °C, electrical power can reach about 4 mW. Figure 14 shows the temperature distribution on the finned tube and the soil moisture content, and the temperature curve is also calculated by Equation (6). Because the experiment was carried out from autumn on, the temperature of the soil below the Earth’s surface decreased with time. All curves trended downward in general, and the calculated *T*_3_ was notably close to the observed *T*_3_. Therefore, the empirical formulas were reliable.

## 4. Conclusions

The finned tube layout is widely used in various ground source heat exchangers because it greatly improves the heat transfer efficiency. This paper studies the spatiotemporal rule of heat transfer on a soil/finned tube surface through simulated analysis and theoretical arithmetic and experimental verification. A copper finned tube with an inner radius of 20 mm, a height of 40 mm, 6 fins and a length of 250 mm, is employed. In the simulated analysis, 36 cases under different working conditions are simulated, and the change processes are obtained. The results indicate that:

(1) The performance of the finned tube is considerably better when the soil moisture content reaches 30%, so any device which harvests heat from shallow soil should be placed where the soil moisture content is greater than 30%; under this circumstance the heat transfer can reach an equilibrium state in 6000 s.
(2) The temperature and heat flux are mainly influenced by soil temperature, soil moisture content and the initial temperature of the finned tube.
(3) Simulations show that the highest heat flux can reach approximately 0.30 mW/mm^2^, and approximately 1507.96 mW of energy can be harvested through the finned tube. A comparison of the simulation and theoretical calculation data shows that the heat transfer rate for this finned tube is 76.77% higher than that of the bare tube if the material is copper.
(4) Two empirical formulas are obtained from the simulation data, and the temperature on the inside of the finned tube and the heat flux absorbed by the finned tube can be obtained using these formulas. The goodness of fit of those two empirical formulas is greater than 97%.
(5) A set of thermoelectric power generation devices was buried in a suburb of Harbin City, and a field experiment was carried out for nine months, where the temperature was continuously observed. The results show that the field experiment data are consistent with the calculated data, and thus, the field test verified the theoretical analysis.

## Figures and Tables

**Table 1:** Thermal properties of soil with changing moisture.

Moisture %	Thermal Conductivity W m^−1^ K^−1^	Specific Heat J kg^−1^ K^−1^	Density kg m^−3^
10	0.3624	731.9	1184.9
15	0.3885	736.7	1178.1
20	0.4125	632.4	1051.6
25	0.4513	601.5	1066.7
30	0.6025	1329.4	1033.5
35	1.2731	659.1	849.3

**Table 2:** Data for *T*_3_ and the heat flux.

*T_s_* °C	*T_f_* °C	Moisture Content (*M*) %	*T*_3_ (°C)		Heat Flux (mW/mm^2^)	
			Simulated	Calculated	Simulated	Calculated
20	15	10	17.39	17.23	0.27	0.27
20	10	10	16.85	16.79	0.22	0.23
20	5	10	16.30	16.35	0.18	0.20
15	10	10	13.43	13.10	0.11	0.11
15	5	10	12.87	12.66	0.08	0.08
10	5	10	9.45	8.98	0.06	0.04
20	15	15	17.48	17.46	0.28	0.27
20	10	15	16.96	17.02	0.23	0.23
20	5	15	16.44	16.58	0.18	0.19
15	10	15	13.48	13.34	0.11	0.11
15	5	15	12.95	12.90	0.07	0.07
10	5	15	9.47	9.21	0.05	0.03
20	15	20	17.48	17.69	0.27	0.27
20	10	20	16.96	17.25	0.23	0.23
20	5	20	16.44	16.81	0.18	0.20
15	10	20	13.48	13.57	0.11	0.11
15	5	20	12.96	13.13	0.08	0.08
10	5	20	9.48	9.44	0.05	0.04
20	15	25	17.56	17.92	0.28	0.28
20	10	25	17.07	17.48	0.23	0.24
20	5	25	16.58	17.04	0.19	0.20
15	10	25	13.54	13.80	0.12	0.12
15	5	25	13.04	13.36	0.07	0.08
10	5	25	9.50	9.67	0.05	0.04
20	15	30	18.24	18.16	0.30	0.29
20	10	30	17.90	17.71	0.27	0.25
20	5	30	17.55	17.27	0.24	0.22
15	10	30	13.95	14.03	0.13	0.13
15	5	30	13.60	13.59	0.11	0.10
10	5	30	9.65	9.90	0.03	0.05
20	15	35	18.68	18.39	0.30	0.31
20	10	35	18.47	17.95	0.28	0.27
20	5	35	18.25	17.50	0.26	0.23
15	10	35	14.24	14.26	0.14	0.15
15	5	35	14.02	13.82	0.12	0.11
10	5	35	9.78	10.14	0.02	0.07

## Nomenclature

*T_i_*	temperature probes
*T_s_*	temperature of soil
*T_f_*	temperature of finned tube
*r*_0_	inner radius of copper tube
*r*_1_	outer radius of copper tube
*r*_2_	radius of 2-D model
*r*	radius
ρ	density of soil
*c*	specific heat of soil
*T*	temperature
τ	time
*λ*	thermal conductivity of soil
*q_v_*	heat produced by soil
*T_a_*	temperature on *r*_1_
*T_b_*	temperature on *r*_2_
*q*	heat flux
*Φ*	heat flow
*L*	length
R^2^	goodness of fit
